# Bath Treatments With Emamectin Benzoate Control Dactylogyrideans Infecting *Colossoma macropomum* Gills Without Altering Physiology of This Host Fish

**DOI:** 10.1111/jfd.70090

**Published:** 2025-12-22

**Authors:** Raimundo Rosemiro de Jesus Baia, Marcela Nunes Videira, Amanda Mendes Pacheco, Eliane Tie Oba Yoshioka, Marcos Tavares‐Dias

**Affiliations:** ^1^ Programa de Pós‐Graduação Em Biodiversidade Tropical (PPGBIO) Universidade Federal do Amapá (UNIFAP) Macapá Brazil; ^2^ Laboratório de Morfofisiologia e Sanidade Animal Universidade do Estado do Amapá (UEAP) Macapá Brazil; ^3^ Embrapa Amapá Macapá Brazil

**Keywords:** blood, *Colossoma macropomum*, dactylogyrideans, emamectin benzoate, fish, gills, treatment

## Abstract

Intensification of fish aquaculture worldwide has led to severe problems of diseases caused by parasitic dactylogyrideans. In this study, the anti‐dactylogyridean efficacy of treating *Colossoma macropomum* with baths of emamectin benzoate (EMB) was investigated for the first time, along with the effects of this treatment on the haematology and histology of its gills. Thirty‐nine fish (three replicates of 13 each) received four consecutive daily baths with EMB at a concentration of 0.5 g L^−1^ and one control group of 39 fish (three replicates of 13 each) only received water from the cultivation tank. The EMB treatments showed effectiveness of 50.2% against *Anacanthorus spathulatus, Notozothecium janauachensis* and *Mymarothecium boegeri* infecting the gills of *C. macropomum* . Fish treated with EMB only caused decreases in the total red blood cell, total thrombocytes, leukocytes, monocytes and neutrophils numbers, while the mean corpuscular haemoglobin concentration (MCHC) increased. Aneurysm was the gill structural alteration caused by EMB treatment, and mean assessment values (MAV) indicated moderate to severe damage due to this irreversible gill alteration. Our results lay a safe foundation for the application of EMB as a potential chemotherapeutic agent for dactylogyridean control in *C. macropomum* aquaculture, because this drug should be used cautiously for anthelminthic treatment in fish.

## 1 Introduction

Aquaculture is undergoing great expansion worldwide because it plays an important role in supplying food to the increasing world population (Yilmaz and Yildiz 2023; FAO 2024). However, the impact of parasitic diseases within aquaculture is also growing, and is becoming a major problem (Abraham et al. 2024). Among the problems for fish aquaculture worldwide are infections by helminthic monopisthocotyleans and polyopisthocotyleans that become pathogenic, leading to massive mortality and morbidity, which give rise to enormous economic losses in production (Yilmaz and Yildiz 2023; Leis et al. 2023; Ávila‐Castillo et al. 2024; Mladineo et al. 2024).

Among these parasitic helminths, dactylogyrideans like *Anacanthorus spathulatus, Notozothecium janauachensis* and *Mymarothecium boegeri* frequently infect the tambaqui, *Colossoma macropomum* (Cuvier, 1818), an Amazonian fish of great importance for aquaculture in this region of South America (Baia et al. 2024). Therefore, treatments to control infections by dactylogyrideans are crucial for ensuring the stable development of fish aquaculture.

Chemotherapeutants such as praziquantel, mebendazole, albendazole, febantel, triclabendazole, formalin, trichlorfon and potassium permanganate have usually been used for controlling these parasitic diseases in fish aquaculture (Reimschuessel et al. 2011; Alves et al. 2019; Oliveira et al. 2019; Leis et al. 2023; Mladineo et al. 2024). However, management and control of these parasitic infections is a constant challenge within fish aquaculture (Mladineo et al. 2024). These actions become greatly complicated by the limited availability of efficacious licensed products and this problem is exacerbated by the development of resistance to antiparasitic drugs in parasite populations. Therefore, there is an urgent need to find new effective chemotherapeutic agents for control and treatment of these ectoparasites within fish aquaculture (Tu et al. 2025).

Emamectin benzoate (EMB) is a semisynthetic avermectin widely used for pest control within agriculture and forestry against nematodes and arthropods (Collymore et al. 2014; Julinta et al. 2020; Abraham et al. 2024, 2025). Currently, EMB is used principally against several ectoparasitic crustaceans in fish. It is added to the diets of these fish because of its effectiveness against all life stages of the crustaceans, its prolonged effect and its ease of administration. Moreover, EMB has been authorised for use in fish aquaculture in several different countries (Julinta et al. 2020; Raja et al. 2022, 2023; Abraham et al. 2024, 2025). However, it has not yet been registered in Brazil.

Nonetheless, given that highly infested fish usually present anorexia, oral treatment during disease outbreaks may not target the most severely parasitized fish. On the other hand, treatments with baths offer an alternative to oral treatments with EMB (Kent et al. 2019). Oral treatments with EMB can cause effects that depend both on the concentration used and on feeding time, which may decrease both the feed intake and the biomass of the fish, thus causing physiological and metabolism problems and fish mortality (Julinta et al. 2020; Das et al. 2023; Abraham et al. 2024, 2025). Therefore, dietary application of EMB in commercial aquaculture must be approached with caution as it may impede fish growth and production indirectly (Abraham et al. 2024). In addition, these treatments leave residues in the fish muscles, depending on the concentrations of EMB used (Julinta et al. 2020; Abraham et al. 2024). However, few studies on treatments with EMB against parasitic helminths have been carried out.

Both oral and bath treatments with EMB (Lice‐Solve) have shown efficacy against the nematode *Pseudocapillaria tomentosa* in *Danio rerio* (Collymore et al. 2014; Kent et al. 2019), and also in in vitro trials against the acanthocephalan *Neoechinorhynchus buttnerae* in *C. macropomum* (Oliveira et al. 2019). Reimschuessel et al. (2011) found that in vitro exposure of *Acolpenteron ureteroecetes* to 10–100 mg L^−1^ of EMB killed these parasitic helminths collected from *Micropterus salmoides* . In contrast, dietary supplementation with 0.015–0.050 mg kgBW^−1^ day^−1^ of EMB had no efficacy against *Sparicotyle chrysophrii*, a helminth Microcotylidae infecting the gills of *Sparus aurata* (Merella et al. 2024).

Recently, we found that EMB (0.5–25 g L^−1^) showed in vitro efficacy against *A. spathulatus* , *N. janauachensis* and *M. boegeri* on the gills of *C. macropomum* , and also that 0.5 mg L^−1^ of EMB was the concentration safest for the host fish (Baia et al. 2024). Given that these results indicated the potential for the use of EMB for controlling these dactylogyrideans of *C. macropomum* , we investigated the efficacy of therapeutic baths with this concentration of EMB against dactylogyrideans on the gills of this host, along with the effects of this treatment on the haematology and gill histopathology of this host fish.

## 2 Materials and Methods

### 2.1 Fish, Acclimatisation and Parasitic Dactylogyrideans

*Colossoma macropomum* fingerlings were purchased from a commercial fish farm in the state of Amapá, Brazil. The fish were transported to the Aquaculture Laboratory of Embrapa Amapá and acclimated for 15 days in a 500‐L tank with constant aeration and continuous water renewal (1.3 L min^−1^). They were fed *ad libitum* twice daily with a diet containing 32% crude protein (Guabi, Brazil). Organic matter from the bottom of the tanks was removed every two days. Previously, 10 fish were examined for the presence of dactylogyrideans (mean intensity of 70 ± 20), and all were found to be parasitized.

The following water parameters were monitored every two days: mean temperature (30.0°C ± 0.1°C), dissolved oxygen (5.8 ± 0.2 mg L^−1^), pH (5.7 ± 0.1), total ammonia (0.4 ± 0.2 mg L^−1^), alkalinity (10.0 ± 0.001 mg L^−1^) and hardness (10.0 ± 0 mg L^−1^).

### 2.2 Obtaining and Composition of the Emamectin Benzoate

Emamectin benzoate (Proclaim 50) containing 50 g kg^−1^ of emamectin benzoate as the active ingredient was used. It was purchased from Syngenta (São Paulo, Brazil).

### 2.3 Treatment of *C. macropomum* With Baths of Emamectin Benzoate

Seventy‐eight specimens of *C. macropomum* (77.5 ± 26.8 g and 16.3 ± 1.9 cm) were used. Thirty‐nine of them were subjected to therapeutic baths with EMB. Our in vitro studies showed that exposure to 12.5 to 25 g L^−1^ of EMB had 100% in vitro efficacy against dactylogyrideans, with variation in time according to concentration. Previous tolerance tests indicated that *C. macropomum* are able to tolerate 0.5 g L^−1^ of EMB in therapeutic baths for up to 2 h. Thus, this concentration was used in therapeutic baths of 2 h per day for four consecutive days (Baia et al. 2024). Every day, this EMB concentration was previously diluted (at a ratio of 0.1 g to 3 m L^−1^ of water) before being added to the experimental tanks.

In this study, the experimental groups were constituted as one control group (0 g L^−1^ of EMB) with water from the cultivation tank, and one group with therapeutic baths using 0.5 g L^−1^ of EMB, with three replicates each (13 fish per replicate), totaling 39 fish per treatment. The fish were kept in a static water system upon addition of the essential oil in the experimental tanks. After this, the water in the tanks was changed.

On the fourth day, following the therapeutic baths, 60 fish were collected randomly and then euthanized by means of medullary sectioning in order to collect gill tissue. This material was fixed in 5% formalin and used to count the parasitic dactylogyrideans, following previous recommendations (Eiras et al. 2006). Then, the prevalence and mean abundance of these parasites were determined (Bush et al. 1997), along with the anti‐dactylogyridean efficacy of the treatments in accordance with the recommendations of Wang et al. (2008).

### 2.4 Histopathology of *C. macropomum* Gills and Blood Parameters After Treatment in Baths With Emamectin Benzoate

At the end of the fourth bath with 0.5 g L^−1^ of EMB, five fish from each replicate (15 fish per treatment) were randomly collected in order to collect blood, to evaluate the blood parameters. A blood sample was drawn from each fish by puncturing the caudal vein using syringes containing ethylenediamine tetra‐acetate (10%). Whole blood was used for the following determinations: haematocrit by the microhematocrit method, total erythrocyte count in a Neubauer chamber, and haemoglobin concentration by the cyanmethemoglobin method. The Wintrobe hematimetric indices of mean corpuscular volume (MCV) and mean corpuscular haemoglobin concentration (MCHC) were calculated. Blood smears were made and panchromatically stained with a May‐Grünwald‐Giemsa‐Wright combination for differential leukocyte counts in up to 200 cells of interest in each blood smear. The identification and nomenclature of the leukocytes, along with the methods for counting total and differential leukocytes, and total thrombocytes were in accordance with the recommendations of Ranzani‐Paiva et al. (2013).

The remaining blood was centrifuged at 75 G (Centrifuga MCD‐2000, Brazil) for 7 min to obtain plasma and determine glucose and total protein levels. Glucose concentration was determined using the enzymatic‐colorimetric glucose oxidase method with a commercial kit (Biotécnica, MG, Brazil). Total protein plasma concentration was determined using the biuret method, via a commercial kit (Biotécnica, MG, Brazil). These biochemical parameters were read in a UV/Visible spectrophotometer (KASVI‐Model 2022/2025) using different wavelengths.

Additionally, another 18 fish were collected randomly and euthanized by means of medullary sectioning and were used to collect gills for other histopathological analyses. The gill arches from three fish from each replicate of the treatments (a total of nine fish per treatment) were collected.

The first gill arch from each side (right and left) was collected and fixed in Davidson's solution for 48 h. Then, the gill arches were dehydrated in a graded ethanol series (70, 80, 90 and 100%) and xylene, and embedded in paraffin to obtain 5 μm slices using a microtome (Easypath EP 31–20,093, Brazil). After mounting on slides (in duplicates), the material was stained with haematoxylin and eosin (HE) and analysed. Images were captured using a digital camera (Moticam 2300 3.0 M Pixel) coupled to a standard optical microscope and a computer. Histopathological changes were analysed semi‐quantitatively using mean assessment values (MAV) (Schwaiger et al. 1997) and the histopathological alteration index (HAI) (Poleksić and Mitrović‐Tutundžić 1994).

### 2.5 Statistical Analysis

The histopathological, parasitological and blood data were evaluated for normality using the Shapiro–Wilk test and the RVAideMemoire package (Herve 2023) and for homoscedasticity using the car package (Fox and Weisberg 2019). The Mann–Whitney (U) test was applied to compare treatments since the data did not present a normal distribution. These analyses were run using the R Core Team software (R Core Team 2024).

## 3 Results

### 3.1 Anti‐Dactylogyridean Efficacy of the Treatment With Emamectin Benzoate

**FIGURE 1:**
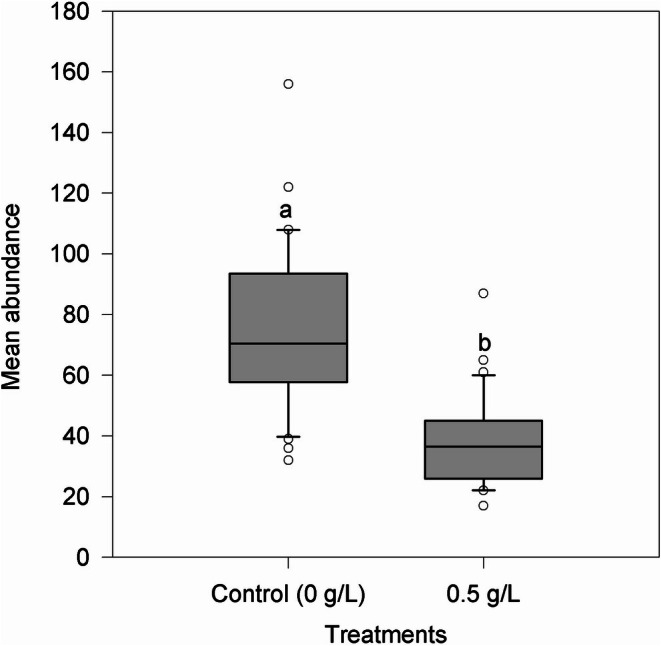
Mean abundance of dactylogyrideans on the gills of *Colossoma macropomum* after treatment with baths containing emamectin benzoate. Data express mean ± standard deviation. Different letters indicate significant differences between treatments.

After four 2‐h baths per day with 0.5 g L^−1^ of EMB, all the fish (100%), in both the treated and untreated groups, were found to present gills infested by *A. spathulatus* , *N. janauachensis* and *M. boegeri*. However, the treatment significantly reduced (*p* < 0.05) the abundance of these ectoparasitic dactylogyrideans (Figure 1), and the treatment efficacy was 50.2%.

### 3.2 Histopathology of *C. macropomum* Gills and Blood Parameters After Treatment With Baths of Emamectin Benzoate

**FIGURE 2:**
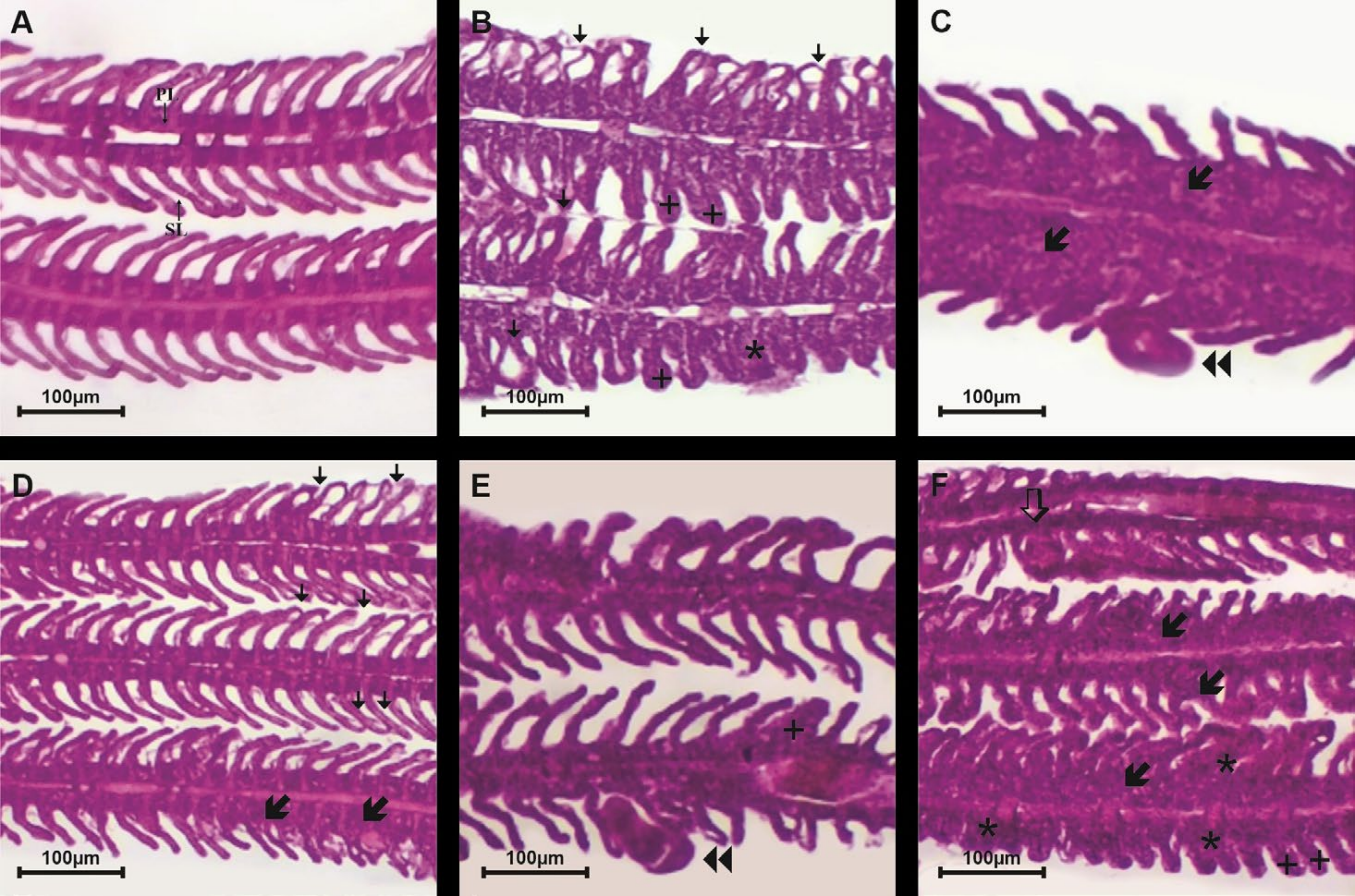
Histological analysis of the gills of *Colossoma macropomum* after treatments with baths containing 0.5 g L^−1^ of emamectin benzoate. (A) Gills of fish of control group exposed to cultivation tank water showing primary (PL) and secondary (SL) lamellae. (B) Detachment of the gill lamellar epithelium (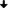), total hyperplasia with fusion of the lamellae (*), lamellar hyperplasia (+) in fish exposed to cultivation tank water (control). (C) Parasite (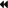) and gill lamellar hyperplasia (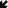) in fish exposed to cultivation tank water (control). (D) Detachment of the lamellar epithelium (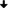) and partial hyperplasia with fusion of the lamellae (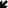) in gills of fish treated with emamectin benzoate. (E) Parasite (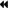) and lamellar hyperplasia (**+**) in gills of fish treated with emamectin benzoate. (**F**) Aneurism (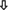), partial hyperplasia (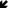) and total hyperplasia with fusion of the lamellae (*), and lamellar hyperplasia (**+**) in gills of fish treated with emamectin benzoate.

On fish gills treated with 0.5 g L^−1^ of EMB and those of untreated fish, alterations such as hyperplasia with partial fusion of the lamellae, hyperplasia with total fusion of the lamellae and detachment of the lamellar epithelium were found. However, aneurysm was the only gill alteration found only in fish treated with EMB. Presence of parasite dactylogyridean was also observed on the gills of both the untreated fish and those treated with EMB (Figure 2).

In the semi‐quantitative evaluation of gill changes, the results showed that the fish treated with 0.5 g L^−1^ of EMB and the untreated fish had similarly high values (*U* = 55.5, *p* = 0.18) for the histological alteration index (HIA). However, the mean assessment values (MAV) (*U* = 64.0, *p* = 0.04) of the treated fish were higher than those of the controls, which were exposed only to cultivation tank water and which had mild to moderate gill damage (Table 1).

**TABLE 1:** Histopathological alteration index (HAI) values and mean assessment values (MAV) for *Colossoma macropomum* gills after treatment with baths containing emamectin benzoate.

Treatments	*N*	MAV	HAI	Severity of lesions according to HAI
Water from cultivation tank	9	11.2 ± 2.2^a^	13.2 ± 3.3^a^	Mild to moderate gill damage
0.5 g L^−1^ of emamectin benzoate	9	14.1 ± 4.2^a^	19.2 ± 5.0^b^	Moderate to severe gill damage

*Note:* Data express mean ± standard deviation. Different letters, in the same column, indicate significant differences between treatments.

In the fish treated with 0.5 g L^−1^ of EMB, the total red blood cell, total thrombocyte, leukocyte, monocyte and neutrophil counts were found to have decreased, while the mean corpuscular haemoglobin concentration (MCHC) increased. The other blood parameters investigated remained unchanged (Table 2).

**TABLE 2:** Blood parameters of *Colossoma macropomum* after treatment with baths containing emamectin benzoate.

Parameters	Control (0 g L^−1^)	0.5 g L^−1^	*U*	p‐value
Glucose (g dL^−1^)	88.5 ± 20.1^a^	100.9 ± 15.4^a^	153.0	0.09
Total protein (mg dL^−1^)	3.2 ± 0.6^a^	3.0 ± 0.4^a^	103.0	0.071
RBC (x 10^6^ μL^−1^)	1.91 ± 0.20^a^	1.50 ± 0.11^b^	6.0	0.001
Haemoglobin (g dL^−1^)	8.1 ± 0.6^a^	7.7 ± 0.7^a^	76.5	0.14
Haematocrit (%)	27.9 ± 2.1^a^	27.8 ± 2.5^a^	115.0	0.93
MCV (fL^−1^)	148.1 ± 22.8^a^	187.0 ± 21.9^b^	204.0	0.001
MCHC (g dL^−1^)	29.1 ± 1.8^a^	27.8 ± 2.8^a^	80.0	0.18
Thrombocytes (μL^−1^)	94,625 ± 14,613^a^	62,099 ± 22,816^b^	24.0	0.001
Leukocytes (μL^−1^)	194, 963 ± 19,887^a^	15,1658 ± 12,055^b^	6.0	0.001
Lymphocytes (μL^−1^)	70,427 ± 9791^a^	70,427 ± 9791^a^	112.5	0.98
Monocytes (μL^−1^)	58,155 ± 18,672^a^	41,574 ± 8545^b^	41.0	0.003
Neutrophils (μL^−1^)	32,721 ± 10,784^a^	25,034 ± 7067^b^	67.0	0.06
Eosinophils (μL^−1^)	1729 ± 1061^a^	2927 ± 1783^a^	151.1	0.11
PAS‐GL (μL^−1^)	16,263 ± 8545^a^	12,678 ± 5770^a^	112.5	0.98

*Note:* Data express mean ± standard deviation. Different letters, in the same column, indicate significant differences between treatments (*p* < 0.05). Abbreviations: MCHC, Mean corpuscular haemoglobin concentration; MCV, Mean corpuscular volume; PAS‐GL, Positive‐PAS granular leukocytes; RBC, Red blood cells.

## 4 Discussion

Different EMB concentrations have been found to present in vitro efficacy against parasitic dactylogyrideans like *A. ureteroecetes* (Reimschuessel et al. 2011) and *A. spathulatus* , *N. janauachensis* and *M. boegeri* (Baia et al. 2024). On the other hand, dietary supplementation with EMB had no efficacy against microcotylidaeans like *S. chrysophrii* on *S. aurata* gills (Merella et al. 2024).

To our knowledge, there are no previous studies on the use of therapeutic baths of EMB to control parasitic monopisthocotyleans or polyopisthocotyleans in other fish species. In this first study, we found that four daily baths with 0.5 g L^−1^ of EMB had 50.2% efficacy against *A. spathulatus* , *N. janauachensis* and *M. boegeri* on the gills of *C. macropomum* . Although this efficacy level is acceptable for controlling ectoparasites according to the World Association for the Advancement of Veterinary Parasitology (Sommerville et al. 2016), this result suggests that more baths with this dosage are needed in order to achieve greater elimination of ectoparasites from the gills of *C. macropomum* . In contrast, a single bath of 24 h with 0.17 or 0.6 mg L^−1^ of EMB completely eliminated the nematode *P. tomentosa* from the intestine of *D. rerio* (Kent et al. 2019). Therefore, our results have provided a new anti‐dactylogyridean chemotherapeutant, along with strategies for controlling these parasites within the aquaculture of *C. macropomum* . However, further studies using treatments with formalin, praziquantel or trichlorfon are needed and also in order to evaluate different times of therapeutic exposure against dactylogyrideans of *C. macropomum* , in comparison with EMB baths. Emamectin benzoate acts on the nervous system of helminths by binding to glutamate‐gated chloride channels (GluCls), increasing chloride ion permeability and causing the nerve cell membrane to hyperpolarize. This disruption of normal nerve signal transmission results in irreversible neuromuscular paralysis and death of helminth parasites (Mallik et al. 2023). However, the mechanisms of action in dactylogyrideans still need to be investigated.

In fish aquaculture, improvement of production and productivity through the use of medicines in fish for human food must always be undertaken in association with concern for any residues that might remain in the meat, given that such residues pose a health hazard to consumers. Although there is a lack of knowledge regarding accumulation of residues from EMB through therapeutic baths administered to fish, studies on residues from dietary supplementation with EMB in fish have been conducted. In *Oreochromis niloticus* supplemented with 50 μg kg^−1^ of EMB for 7 days, the peak residue levels in the muscles were found after the first day of feeding, and the levels decreased subsequently and remained at levels within the acceptable limits for human consumption (Julinta et al. 2020). Similarly, Abraham et al. (2024) reported that in *O. niloticus* , 21 days after interruption of feeding with 50–500 μg kg^−1^ of EMB for 14 days, residue traces were detected in muscles.

Emamectin benzoate has long‐acting antiparasitic action on crustacean parasites and protracted tailing‐off periods when the active component concentration in fish flesh falls below optimum levels. Since EMB is a potent neurotoxic agent with a half‐life of 9–11 days in fish muscle tissue, plasma and mucus, the European Medicines Evaluation Committee has set the maximum residue limit for EMB at 100 μg kg^−1^ in fish fillets for human consumption, with an acceptable daily intake of 0–0.005 mg kg^−1^ of biomass in fish (Abraham et al. 2025). Although such information about the bioaccumulation process of EMB in fish meat and its half‐life in therapeutic baths remains unavailable, it can be presumed that a safety margin of 30 days after these treatments should be respected in order to ensure consumer health.

In the gills of both untreated *C. macropomum* and the fish treated with 0.5 g L^−1^ of EMB, hyperplasia with partial fusion of the lamellae, hyperplasia with total fusion of the lamellae and detachment of the lamellar epithelium were the alterations observed. However, aneurysm occurred only in the gills of the treated fish, which presented moderate to severe gill damage: this is irreversible structural damage to this respiratory organ in fish. In contrast, one bath treatment with 0.17 or 0.6 mg L^−1^ of EMB for 24 h did not cause structural damage to the gills of *D. rerio* (Kent et al. 2019), while our results showed that the gill epithelium of *C. macropomum* was highly sensitive to consecutive treatments with this higher concentration of EMB. Therefore, since the gill epithelium is the respiratory organ of fish, serving as their primary interface of contact with any chemical exposure, caution needs to be taken regarding the use of EMB for treatment with baths for each host fish species.

Although EMB is a crucial chemotherapeutant in fish aquaculture for controlling and treating diseases caused by different ectoparasitic crustacean species, its use can lead to disruption of the physiological and blood biochemical processes in fish fed with or exposed to this agent. The toxic effects of EMB in fish are influenced by several factors such as concentration, duration of exposure, bioaccumulation, species, age, size, sex, water quality parameters and type of pesticide formulation, along with EMB absorption, accumulation, metabolism, excretion and degradation (Kumar et al. 2022). Dietary supplementation for *O. niloticus* consisting of 50–500 μg of EMB kg^−1^ of biomass per day has been shown to cause alterations (increase or decrease) in haematocrit, mean cell haemoglobin concentration (MCHC), total erythrocyte, leukocyte, thrombocyte, monocyte, lymphocyte and neutrophil counts, haemoglobin levels and glucose levels (Das et al. 2023; Abraham et al. 2024, 2025). These alterations are due to toxicity accompanied by hepatomegaly and splenomegaly (Abraham et al. 2024) in both haematopoietic organs. Therefore, these results demonstrate the direct stressful effects of dietary EMB at the recommended and higher doses, on the haematological parameters and vital organ functioning of *O. niloticus* (Abraham et al. 2024).

In *C. macropomum* , four therapeutic baths with 0.5 g L^−1^ of EMB decreased the total red blood cell, thrombocyte, leukocyte, monocyte and neutrophil counts while increasing the MCHC, which seems to be an adaptive response to exposure to this low therapeutic concentration. In *Labeo rohita* , for which the lethal concentration (LC_50‐96h_) of EMB was found to be 91.0 μg L^−1^, exposure to a sublethal concentration of 9.1 μg L^−1^ caused decreases in haemoglobin levels, haematocrit and total red blood cell and leukocyte counts, with an increase in MCHC (Kumar et al. 2022). Thus, these were small physiological alterations, compared with those reported in previous studies on *O. niloticus* (Das et al. 2023; Abraham et al. 2024, 2025). Additionally, studies have demonstrated that blood parameters are reliable biomarkers of induced stress due to EMB (Kumar et al. 2022; Abraham et al. 2024), as well as of toxicity. Therefore, complementary assessments such as blood chemistry (e.g., in relation to gill, liver and kidney function) and histopathology of the liver, kidney and spleen should be considered to ensure that EMB treatment does not adversely affect fish health. Such assessments would support its safe application in fish aquaculture practices.

## 5 Conclusions

The results demonstrated that four baths with 0.5 g L^−1^ of EMB did not cause significant blood changes in *C. macropomum* , although this led to gill aneurysm, thus indicating toxicity signs. In addition, the results demonstrated that this treatment strategy with baths had significant effectiveness against dactylogyrideans, and suggested that the efficacy might be improved by increasing the number of days of baths to 6–7 days. We showed that EMB can control infections by *A. spathulatus* , *N*. *janauachensis* and *M. boegeri* in *C. macropomum* . Hence, our study lays the foundation for the application of EMB as a new chemotherapeutic drug for fish aquaculture; however, it should be used with caution.

## Author Contributions

**Raimundo Rosemiro de Jesus Baia:** formal analysis, data curation, writing, writing‐original draft; **Marcela Nunes Videira:** methodology writing – original draft, supervision; **Amanda Mendes Pacheco:** methodology, formal analysis; **Eliane Tie Oba Yoshioka:** writing – original draft, formal analysis; **Marcos Tavares‐Dias:** corresponding author, submitting author, financing, conceptualization, investigation, review and editing.

## Funding

This work was supported by Conselho Nacional de Desenvolvimento Científico e Tecnológico, 301911/2022‐3.

## Ethics Statement

This study was developed in accordance with the principles adopted by the Brazilian College of Animal Experimentation (COBEA) and with authorization from the Ethics Committee for Use of Animals of Embrapa Amapá (Protocol No. 021: CEUA‐ Embrapa Amapá).

## Conflicts of Interest

The authors declare no conflicts of interest.

## Data Availability

The data that support the findings of this study are available on request from the corresponding author. The data are not publicly available due to privacy or ethical restrictions.
